# Placement Architectures in Practice: An Exploration of Student Learning during Non-Traditional Work-Integrated Learning in Rural Communities

**DOI:** 10.3390/ijerph192416933

**Published:** 2022-12-16

**Authors:** Elyce Green, Sarah Hyde, Rebecca Barry, Brent Smith, Claire Ellen Seaman, Jayne Lawrence

**Affiliations:** 1 Three Rivers Department of Rural Health, Charles Sturt University, Wagga Wagga, NSW 2678, Australia; 2 Joint Program in Medicine School of Rural Medicine, Charles Sturt University, Orange, NSW 2800, Australia; 3 School of Nursing, Paramedicine and Healthcare Sciences, Charles Sturt University, Dubbo, NSW 2830, Australia

**Keywords:** work-integrated learning, Theory of Practice Architectures, rural health, clinical placement, allied health, student learning

## Abstract

Background: Work-integrated learning (WIL) in rural communities provides students with important learning opportunities while also providing a service to those communities. To optimise the potential benefits of work-integrated learning for health students and rural communities it is important to explore the practices and outcomes of these experiences. Methods: This study used a qualitative research design underpinned by the theoretical framework of Theory of Practice Architectures to examine the way students learn during these placements. Purposive sampling was used to identify students for participation in the study. Seven students from the disciplines of paramedicine, physiotherapy, and speech pathology participated in semi-structured interviews. Data were analysed using inductive thematic analysis. Results: The learning described by the students was examined, followed by a critical interrogation of the data to assess how these learnings and associated practices were made possible given the site-specific practice architectures. The findings of the research are represented by three themes: learning affordances related to placement design, learning through relationships between people and professions, and learning through rural embeddedness. Conclusion: Being embedded in rural communities gave the students access to several arrangements that fostered learning, particularly through the sayings, relatings and doings that the students engaged with. This research demonstrates the transformative potential of rural WIL opportunities for learning and future rural practice.

## 1. Introduction

Work-integrated learning (WIL) provides students with opportunities to apply and develop their professional knowledge in different practice settings to build their capacity to function as a professional in analogous practice contexts [1]. WIL is facilitated by tertiary institutions as part of their pedagogical mandate to produce work-ready graduates who can meet core professional practice requirements [1]. In this sense, WIL is expected to be transformative; to support students’ professional becoming by situating them in applied occupational contexts where they can engage in relevant professional practices [2,3]. Importantly, WIL experiences also support broader professional becomings through enabling students to identify, engage with, and assess different possibilities for practising as a professional [4]. This could include working with different population groups, with different professional purposes, and in specific practice settings. Through engaging and, in some way, realising these possibilities, students can also contribute to and transform practices in these sites. Through a TPA lens, WIL therefore presents the potential for creating social change through coming to practice differently [4,5,6].

It is this transformative potential that is a central tenet of efforts to increase high-quality WIL experiences in underserved communities. There is the expectation that WIL experiences will enable students to build the knowledge of, interest in, and capacity to work with communities with workforce shortages, such as in rural areas, and thereby come to contribute to health practice in those settings. There is, however, a need to better understand the local contexts and social processes—the learnings, practices, and supportive practice conditions—that contribute to the potential of rural WIL experiences to positively influence rural workforce recruitment efforts [7,8]. Current literature demonstrates the limited understanding of what constitutes a high-quality WIL experience in rural areas, and there is a recognised need to further explore how students can be supported to engage in and learn from these opportunities [8].Developing a better understanding of how students learn the practices of being a health professional serving rural communities, and interrogating the transformative potential of these placements, is needed to inform ongoing quality improvement in rural health placements as well as the policies that dictate key performance indicators for universities [8]. 

Addressing this need, this research used qualitative methods to explore the learning experiences of students from health disciplines undertaking non-traditional rural placements with specific population groups. This paper considers student placements as a specific form of WIL, defined as an unpaid period in which a student attends an approved professional workplace as a requirement of the course they are studying [9]. In particular, this research explored the factors which shaped student learning and their emergent professionalism in these unique settings. The Theory of Practice Architectures (TPA) [6,10,11] framed the design and investigation, and informed the findings related to student learning in these rural placements. The findings of this research can inform how to prepare students and supervisors for these placements into the future, as well as indicating how universities can best support students, supervisors, host sites, and communities during placements more broadly. It is intended that this research will help to inform the building of sustainable partnerships with rural communities and health providers to ultimately provide high-quality student learning experiences.

### 1.1. Theoretical Framework

The Theory of Practice Architectures (TPA) was used to frame the methods and data collection for this research, and as a lens from which to understand the findings. Ontologically, the TPA is concerned with what practices are, how they happen, and what shapes, constrains, and enables practices [6] (p. 17); and epistemologically, how we can learn in practice [6] (pp. 17–18). Practices refer to ‘sayings, doings, and relatings that hang together’ in a coherent way as part of the pursuit of projects which individuals are motivated to undertake for some expected outcomes [6] (p. 8). Consistent with practice-based theories, TPA views individual sayings, doings, and relatings in the intersubjective space as dialectically produced and re-producing the socio-political, cultural-discursive, and economic-material arrangements that make such practices possible [11] (p. 242). The TPA is useful as a ‘transformational resource’ for informing educational practice [6] (p. 2). This is, in part, because the TPA supports analysing the conditions of possibility that enable and constrain various practices across different sites. This can be used to make practical judgements to support arrangements that hold those practices in place to enable positive, transformational goals [6] (p. 20).

Cultural-discursive arrangements refer to the resources that make sayings in a practice possible. These sayings inform the thinking, performing, and justification of a practice [6]. Material-economic arrangements refer to aspects of the physical environment that shape the doings of a practice by affecting how, when, and by whom something can be done [6]. Social-political arrangements refer to the resources that shape relatings in a practice to one another and to non-human objects [6].

The situatedness of learning and practices means that the sayings, doings, and relatings occur in an intersubjective space, where the person and the site are inextricably linked [10,12]. As such, the TPA acknowledges that individuals come to practice with their own projects and ways of practice [12]. This is useful because of the role that individuals and their own personal epistemologies have in determining how, whether and what practices for learning are engaged with [2,13]. Personal epistemologies are “active, intentional, and derived in person-particular ways through the unique set of socially derived experiences that comprise individuals’ life histories or ontogenies” [13] (p. 210).

In the current paper, we view praxis-orientation as a dynamic component of an individual’s personal epistemology. As the TPA fundamentally seeks to understand how to support development of individual and collective praxis [4,6]—a moral, socio-historically situated, commitment towards acting in accordance with perceived good consequences for others [14]—the theory is highly relevant to studying the learning experiences of health students on WIL experiences developed through and for rural communities. It also supports the centering of the drive of health workers to benefit others, including specific population groups such as their local community or in rural areas more broadly; the underlying rationale for the development of the TRPM WIL experiences.

To date, the TPA has been used primarily in educational contexts to investigate the practices of teacher education and teaching from early childhood through to special education and teaching in vocational and tertiary education, instructional leadership, and curriculum renewal [12,15,16,17,18,19,20,21]. Outside of teacher education, the TPA has been used to understand nursing education and practice, simulation in health professions education, research practices, climate change, and engineering education [5,22,23,24]. Few studies have examined practices concurrently at multiple sites across different discipline areas. The application of the TPA to research focused on the experiences of health student learning in rural WIL has received little attention. This study takes an approach similar to that of Penman et al. [25] who explored feedback practices within allied health clinical placements. The authors utilised the TPA in their study to extend the frame of feedback to cover the conditions and influences that contributed to feedback occurring in a certain way, rather than simply identifying who gave feedback and how [25].

The TPA is an appropriate lens to examine TRPM placement experiences because these placements are designed to provide differing conditions of possibility for practices and therefore of student learning, relative to traditional placement models. It was expected that students’ experiences would be distinct across sites, as in any placement (or practice site), yet they would also share commonalities afforded through distinct conditions of possibility, particularly conditions that demarcate these non-traditional learning experiences from others.

### 1.2. Context

In the context of international tertiary education, WIL is imperative for the preparation of future health professions graduates. In Australia, health professions students are required to undertake tertiary education inclusive of WIL to be able to graduate and be eligible for registration as a health professional. The importance of WIL as a mechanism to learn practice is related to the unique capacities of WIL which allow students to practice and learn within socially complex workplaces [1].

Rural health is one underserved practice setting that relies on WIL experiences as a method of attracting future workforce. Fundamental to this is the expectation that positive, rurally based WIL experiences can influence students’ rural practice intentions [26]. There is evidence that rural WIL experiences encourage allied health and nursing students into future rural work, however, there is limited understanding of the mechanisms through which this occurs, and what best practice looks like for health students outside of medicine [26]. Currently in the Australian context, system-wide health workforce shortages are also driving the demand for training [27], while the need for students to experience WIL is putting increasing pressure on a stretched system where there are few supervisors available [28]. There is a need for creative and innovative solutions to meet WIL demand [27,28], especially in rural health settings where WIL is viewed as a vital pipeline for building the rural health workforce [26].

The Three Rivers Placement Model (TRPM) is a non-traditional placement model designed in response to this need for innovation. In this context, ‘non-traditional’ WIL refers to WIL experiences that do not utilise the apprentice style of training [29]. The TRPM is a WIL programme that focuses on health student placements in rural Australia and was initially developed with a praxis orientation “to increase the capacity for placements in rural and remote areas, contribute to rural workforce, develop supervisory models, and respond to community needs” [30] (p. 286).

This research explored the experiences of a cohort of students undertaking placement within the TRPM and the effect of the TRPM on students’ learning. The TRPM placements were all undertaken in rural communities, defined as areas outside of major cities under the Modified Monash Model which characterises Australian geographic areas based on population and relative access to services [31]. Rural health student placements make an important contribution to WIL programmes internationally by boosting WIL numbers, servicing rural communities, contributing to students’ understanding of the complex relationship between socio-geographic location and health, and developing the future health workforce in these communities [32,33,34]. The TRPM placements focus on students working with specific population groups in a way that supports interprofessional learning opportunities and fosters respect for health consumers. These placements can be initiated by rural communities and are often shaped through community co-design and collaboration principles.

Under the TRPM, students from a range of allied health and nursing courses are typically placed in pairs within a host organisation for time periods of either three, five, or eight weeks depending on curriculum requirements. The TRPM requires the completion of pre-placement learning modules tailored to the rural location and participation in a briefing via a virtual meeting room to prepare students for the unique nature of these placements. The purpose of exploring student learning during the TRPM was to understand the mechanisms by which these placements can be enhanced, particularly in relation to the student learning experience and the associated transformative potential of the placement.

### 1.3. Research Questions

This study addressed three research questions:

1. How does placement design affect student learning?
2. What are the relationships between learning and practice during a rural health student placement?
3. What arrangements enable or constrain practices that contribute to student learning?

### 1.4. Ethical Approval

The study was approved by the Charles Sturt University’s Human Research Ethics Committee [Protocol number: H20262].

## 2. Methods

### 2.1. Design

This study used a qualitative research design underpinned by the theoretical framework of the TPA. Semi-structured interviews were conducted by two authors (CES & SH). They both have a background in tertiary education, health education research, and conducting qualitative research but are not registered health professionals. SH was the Rural Health Lead whose team coordinated the TRPM placements but was not the primary contact for students on placement. CES was the Programme Evaluator for the coordinating department and had no direct contact with students outside of the interviews. Their relationship to the project and project staff was made clear at the commencement of the interviews.

### 2.2. Participant Selection

Purposive sampling was used to identify students for participation in the study. Students participating in the placements under the TRPM were briefed online prior to the placement starting via a virtual meeting room to provide an overview of the placement and inform them about the research. Students were informed that this was a new placement initiative for which feedback was being sought and that they would receive an email with a letter, participant information statement, and consent form attached inviting them to voluntarily participate in this study. An invitation was sent to the students by email two weeks prior to the end of the placement.

Fifteen students from the disciplines of paramedicine, physiotherapy, and speech pathology were invited to participate in the research. Students who responded to the email and consented to participate in this study attended an individual interview or an interview in pairs (two students, one interviewer), according to their preference. Participants were given the option to be interviewed in pairs as they were all undertaking their placement in pairs and the interviewers felt that this option may decrease the risk of discomfort during the interviews. Interviews were conducted face-to-face, via a virtual meeting room, or telephone within two months of placement completion. All students who nominated to participate in the research followed through to interview (none withdrew).

### 2.3. Data Collection

Drawing on the work of Mahon and colleagues [6], the TPA was used to orientate the interviews and frame the data collection. The interviews were semi-structured around a schedule (sample questions shown in Appendix A) consisting of items that would deliberately provide information about the students’ learning, associated practices, and conditions that enabled or constrained different practice at each site as well as student perceptions of the population, the service, and the location, perceptions of support and feedback about the organisation of the placement. The interview guide was piloted with eight students prior to the research commencing. The setting and time of each interview was determined by the preference of the interviewees. Interviews that were conducted in person were completed at the students’ placement site in a private room. Permission was asked to audio-record the interview for transcription purposes only. No compensation was provided for participation. During the interviews, the interviewer took notes and recorded observations to the extent that the interview medium allowed. No non-participants were present during any of the interviews. Participants were offered the opportunity to read through transcripts after interviews were completed, however all participants declined.

### 2.4. Data Analysis

Five members of the research team (EG, BS, CES, JL, RB) conducted inductive thematic data analysis following the process outlined by Braun and Clarke [35] including familiarisation, initial coding, comparison of codes and creating themes, reviewing themes, and defining and naming themes. The researchers met in person to conduct intensive analysis sessions. Each member of the analysis team read two successive transcripts, discussed potential codes, and then created a code book to guide subsequent coding. Next, each researcher was allocated three transcripts and coded as per the code book. The researchers met several times during this process to discuss codes and add to the code list. Coding was conducted manually and each of the researchers linked codes to data extracts. Once all transcripts had been coded, a sixth researcher (SH), who had been involved in the data collection and study design, was assigned two transcripts for independent coding. This sixth researcher had no prior knowledge of the previous codes generated by the initial five researchers. The codes generated by the sixth researcher were aligned with the codes generated by the initial five researchers and informed the final codes.

The final codes were sorted into potential sub-themes and themes across a mind map and the researchers discussed whether the meaning of the transcripts had been adequately represented. Each theme was defined through discussion and the final thematic map was agreed on by all researchers. Once the thematic map was complete, the TPA lens was applied to the data and themes were re-examined for evidence of practices in the semantic, social, and physical intersubjective spaces which enabled or constrained practices for learning. These were then further interrogated to identify the prefiguring arrangements of these practices across the TRPM sites.

### 2.5. Trustworthiness of the Study

This qualitative research was designed with the intention to provide trustworthiness in the results by using design qualities that increased the study’s credibility, transferability, dependability, and confirmability [36]. The credibility of the results of this study was demonstrated by the ongoing peer review between the six researchers who analysed the data and matching codes directly to participant excerpts. The authors have addressed the transferability of the research in the limitations section of this paper and reference to a thorough description of the placement design is given to allow readers to determine the transferability of the results to different contexts. To demonstrate dependability, the researchers kept detailed documentation regarding the process of forming the initial codes through to creating the final themes and linked participant excerpts directly to their thematic representations in the results section of the paper. This process combined with the reflexivity of the researchers as they peer reviewed their data analysis contributes to the confirmability of the study. To further increase the trustworthiness of the study, the researchers have addressed all 32 consolidated criteria for reporting qualitative research suggested by Tong et al. [36].

## 3. Results

Of the 15 students who were invited to participate in the research, seven opted to be involved and were interviewed. The seven participants were from the disciplines of paramedicine (*n* = 3), physiotherapy (*n* = 3), and speech pathology (*n* = 1). The interviews were all conducted in 2020 and 2021 and ranged from 30–60 min. A description of the placement sites, type of placement, and participant characteristics of those involved in this research is included in Table 1.

In relation to the research questions, thematic analysis of the interview transcripts identified student learnings including professional skills such as clinical skills and interprofessional practice related to participating in a non-traditional placement model that promoted autonomy, working with multidisciplinary teams, and being embedded in rural communities. They also reported experiences of developing their professional identity, growing confidence, practising collaboration, and feeling valued.

The results from the initial analysis were then critically interrogated to assess how these learnings and associated practices were made possible given the site-specific practice architectures. Particular attention was paid to the socio-political arrangements and manifest practices, including the influence of these on students’ development of praxis. From this approach to the analysis, the findings of the research are represented by three themes: learning affordances related to placement design, learning through relationships between people and professions, and learning through rural embeddedness.

### 3.1. Learnings Afforded by Placement Design

Across the different placement types, the students noted that they were often required to work autonomously with less direct supervisory oversight. They described how this allowed them to practice their skills and grow their confidence as a health professional, for example, one student commented:

This placement we really needed to set our own deadlines, kind of set our own expectations and hold ourselves accountable. Like there wasn’t someone there like, ‘come on you need to have this by this day’. Like, we weren’t sort of—we didn’t have our hand held as much as we had in prior experiences in placement. So, it was great because it pushed us to work on those skills of just getting ourselves and just hooking in.

(Speech pathology student)

The student autonomy required within the placement also empowered the students to feel ownership over the outcomes and gave them a sense that they made a valuable contribution:

… it’s not often you get an opportunity in a placement to develop a programme and be able to actually have an impact and leave something behind when you finish.

(Physiotherapy student 2)

Evident across the student experiences is that the autonomous learning opportunities were made accessible through a supportive workplace culture and site relations, as will be further discussed below. A key material facilitating arrangement of autonomous learning was that some of the host organisations, particularly the mental health and disability services, had periods of decreased activity. The students noted that the non-traditional arrangements of the placements, particularly those who experienced service delivery downtime, enabled them to proactively examine their own learning goals and seek out opportunities to learn more about the specific client group with whom they were working. This is evident in the following statement from a paramedicine student:

It’s so different to what I’m used to…for a while there I was a bit frustrated because of the slower pace. It’s not an ambulance so I can’t do all the skills that I’m used to doing, which I love doing. So, I’m like, ‘OK, what do I love about paramedics?’, and I was like, ‘Oh, I love interacting with people’, [and] so, I’m like, ‘OK, let’s learn all about that’. […] I feel like, [other] students could go one of both ways. They could either embrace it completely or they could completely be like, this is a waste of time.

(Paramedicine student 1)

This also highlights that while the placement design had a positive effect on students’ learning, the design may constrain learning opportunities for other students who prefer more traditional models and do not reflexively engage in their learning. A key placement design factor which enabled innovative practices for learning to be realised therefore also includes the selection of students who have the capacity to learn in this unique model.

Supporting students to be in this mindset and to focus on the placement opportunity was facilitated by material-economic, socio-political, and cultural-discursive placement preparations. Accommodation support or the ability to live at home during the placement, support from the coordinating clinical educator, and pre-placement online learning modules were described by the students as supporting their capacity to engage with the unique learning opportunity.

…all the accommodation was sorted, with plenty of time. With like a month or so, or two months or whatever it was. And yeh all this like [the placement facilitators] were totally chill and like oh if you need any help, flick us an email, sort of thing, ring us whatever. There were the zoom meetings, there was the um information modules, or training modules, there were all sorts of different things to do. Whereas in comparison to my other placement, that doesn’t always happen.

(Paramedicine student 1)

In some settings, the non-traditional arrangements of the placements, such as altered or reduced direct supervisory oversight and non-clinical practice contexts, constrained opportunities for students to demonstrate their application and development of their more technical clinical skills which form the basis of their placement assessment. This presented some challenges for their clinical supervisors, indicating that future non-traditional placement design should foreground these requirements and how they could be interpreted and demonstrated specific to the placement context. One of the speech pathology students acknowledged this:

It was a bit tricky to really fit our placement to the standard competencies we needed to meet. They were like oh a lot of the competencies in our grading system are for us just seeing individual clients like traditionally so when it came to our placement, […] at first, [the clinical supervisor] was finding it a bit tricky to kind of see how our stuff applied to those competencies.

(Speech pathology student)

Overall, this theme demonstrates the prefiguring effects of the placement design had on the students’ learning, contingent also on students’ own reflexive capabilities to identify and engage with emergent learning opportunities.

### 3.2. Learning through Relationships between People and Professions

The students commonly described a socio-political arrangement that was fundamental to the opportunities for autonomous, student-led learning during placement: being treated as a valued member of their placement organisation. This occurred through interrelated sayings, doings, and relatings from the organisation and the community, for example:

The first day, everyone said hi, everyone introduced themselves. We were given our own desk in the office with them, all the other health professionals. The health professionals on a Friday would have [an office gathering] and we were invited to join and eat along with them which was nice. We had our own email addresses, so we were emailed into everything that was happening. Just really felt a part of that team during those five weeks, rather than just, yeah, I didn’t feel as much as a student. I know a lot of people on placement, just, we’re referred to as “the students”. So it was nice to actually be called, you know [my name].

(Physiotherapy student 1)

These practices mutually reinforced trust and respect with students which positively impacted their opportunities for collaborative learning and building of shared understandings of site-specific practices. Specifically, it impacted learning through supporting the students to build effective working relationships and an understanding of the value of other staff and health professionals in the organisation, and thereby situate their own contribution and learning opportunities to meet the autonomy demands of the placement design. One student felt that:

If [the host organisation staff] were wondering why we were doing something they would just be like, ‘look, we don’t have knowledge, can you let us know why you’re doing this and why/how it will benefit the kids etc’. So yeh, although they didn’t have a good knowledge of what physios do for children, they were willing and open to try new things and learn about why we were there and how we can help the children. […] But also, they gave us ways that we can interact with the kids so if some of them weren’t engaging with us, we’d go and ask them….

(Physiotherapy student 2)

Through positive, respectful relationships with other professionals, as well as clients and the community, these placements supported students to build their professional identity and practice confidence. The student experiences illustrated how these arrangements made possible practices for learning such as shadowing, applying clinical reasoning and delivering professional opinions, seeking clinical and cultural advice, and building confidence engaging in the discursive practices of the client group to better meet their service needs. This was further supported through the co-location of several health professionals within a single site of service delivery, or cross-collaboration between organisations, which enabled interprofessional teamwork and cross-disciplinary collaboration, shown by the following comment made by a student:

I refer back to that placement being something that really pushed me to the next level and I learnt how to collaborate with an organisation and complete consultation work and just really gain a better understanding and appreciation of working with a group of people for a combined outcome, like for overall outcome.

(Speech pathology student 1)

Learning through authentic professional practice with others was also enabled by the students relating with their supervisors and other staff. Supervision was perceived to be particularly effective for learning when the students felt the supervisor was tuned into their learning needs and communicated accordingly, reflected in the following statement:

A fantastic supervisor, lots of experience so very lucky to have her and she was one of the few supervisors who really adapted her supervision, depending on your personality. Like she could pick up on, you know, just how I preferred more concurrent feedback just like straight after as I’m more of a visual learner. So she helped me with that and like using resources and stuff like that, yeah. So I was very lucky.

(Physiotherapy student 1)

The respectful working relationships also supported students to learn and engage in the specific cultural-discursive practices of the organisation and client groups. In some instances, the distinctiveness of the discursive practices of some client groups served to constrain students’ practice capacities and further engagement with clients until they learnt and were able to participate in appropriate practices.

I found it challenging just with the limited knowledge that I had on drugs and alcohol as well as mental health […] just those basic things like when someone came in and said they smoked a certain amount of cones or something a day, I was like, I don’t know what a cone is […], that limited knowledge held me back in that first week, but once I understood, like that was less challenging then.

(Paramedicine student 2)

Despite identifying a lack of prior knowledge of drug and alcohol cultural-discursive practices, the student was able to learn through their relations with their supervisor and consumers, which, when combined with the material conditions of the placement (such as time), and the respectful relations fostered by the supervisor, meant that they could develop a respectful praxis for working with key client groups in the future.

### 3.3. Learning through Rural Embeddedness

Rural embeddedness refers to practice opportunities beyond the geographic space. Relationships fostered in the placement locations were situated in and influenced by the rural context. The capacity to build local relationships and the associated placement arrangements were vital to perceptions of rural embeddedness and students’ learning of the local area. In relation to this theme, one student explained:

So, [the placement organisation] was more like, community-based, which was nice. So, getting to build those relationships with people. In [other rural placement location], at a hospital—I wouldn’t really say it was rural. Like, in terms of when I think of rural, I think of, you know, your client, you know; their family and like you have that relationship. In [other rural placement location], I didn’t get to develop those relationships with my clients.

(Physiotherapy student 1)

The pre-placement preparation, followed by the immersive experience geared students to better understand the potential for future rural living and working, and allowed them to compare this to their previous experiences and other potential practice opportunities in less rural locations.

I just felt the combination of speaking to people [during placement] and even the [organisation] modules that we did […] highlighted the inequity and access to services and things like that. So, learning that through the modules and then seeing the difference between working in [regional centre] versus at home when I am just working at the clinic [in major city area], I could then see that in real life. It was good to have that experience in [regional area] and it kind of made me want to go back. […] I think working rurally as a clinician would require you to be a lot more independent and almost driven to really almost understand everything and everyone that walks in the door you are going to be able to serve them and support them […]. Like you are it and you need to make sure you keep levelling up and you can help everybody, but I think that’s fantastic for professional development.

(Speech pathology student 1)

As described in the placement design, rural embeddedness was facilitated through placement arrangements that supported students to have informal conversations, ongoing client relationships, and building rapport with local staff, clients, and broader community. This not only gave them a well-rounded understanding of clients’ diverse experiences and health needs but enabled them to become more familiar with local arrangements and practice opportunities which they could assess against their own personal and professional goals. For example,

It’s a small community so, for example, the ambulance station was just around the corner from [placement name] and one of the paramedics was actually on the Board. So, as a paramedic, that shows me that there’s options to get involved with the community… So, it’s like, its more than just a little town. It offers so much more…so much interesting stuff.

(Paramedicine student 1)

For students placed with the mental health and disability services, the potential of rural practice related more to knowing the community and seeing the benefits of local living, rather than giving them a taste of the ways practising rurally can be impactful as well as high-pressure. This is likely related to the type of placement setting, in that the health services provided were outside of their typical disciplinary professional practice. One student explained:

We would do day trips around [rural town]. So, it was good; I got to see [the town] through those day trips with the residents and my preceptor. […] I actually want to go back out there with a bunch of friends and show them those areas, ‘cause they’re really pretty.

(Paramedicine student 2)

Comparatively, students who undertook service-learning placements more specifically described changes to their understandings of practice as a rural professional, as well as their consideration of working with the client population in the future. This occurred through practice opportunities that enabled learning more about the need for increased health service provision relative to other practice potentials, as well as having first-hand experience in achieving positive impacts during placement.

I think just going rurally and you see the need for the services, and you get to have a greater impact, I think. People are really needing services out there and as a clinician or someone that goes into a profession like this you go into it to help people, and you want to help everyone, but to see there’s a community that’s in more need of your services you feel well if I could put myself in that community and do that then I would want to.

(Speech pathology student 1)

One student who was interviewed after graduating reported that the learnings and relationships from this placement motivated their choice of graduate work location and continues to inform their professional practice. The student was able to carry these relations to their practice at another, more remote site. The combination of rural, community-organisation, autonomy, and experience working with clients with specific needs has supported the student to become a practitioner in a more remote location.

I feel like, even now—not that I’ve been in my job for long—but that placement has really given me the confidence to work with children. So now I’m getting referrals for children and now I’m like, yeah, I feel more comfortable in this space. Now I know where to start. And I’ve also got the network from that placement as well. So, my supervisor, I still have her email and she said, feel free to contact me. So yeah, I just feel a little more supported outside as well, having graduated with that paediatric placement.

(Physiotherapy student 1)

Overall, the students’ feelings of rural embeddedness were facilitated through placement arrangements that supported students to have informal conversations, ongoing client relationships, and building rapport. This enabled the students to become more familiar with the sayings and doings of the local, rural cultures.

## 4. Discussion

This research demonstrated several affordances related to designing and delivering rural health student placements that enhance practice opportunities for students’ learning of, and contribution to, rural and professional practices. These affordances are interpreted through the application of the TPA as enabling or constraining arrangements that supported student learning. In this way, the results move beyond identifying the skills that can be learnt during a placement and show some of the mechanisms that can influence how learning occurs.

For the TRPM placements, learning affordances were related to the placement design factors, relationships between people and professions, and being embedded in a rural community. While the activities engaged in by students at each placement site varied, through the TRPM design, students described similar approaches to sayings, doings, and relatings that positively contributed to similar transformations in their future practice intentions. The activities also increased the students’ interest in and capacity for health practice with specific client groups.

By applying the TPA, this study has shown the importance of students perceiving the contributory effects of their participation in their professional becoming. Implicit in the participating students’ accounts of their learning is the pursuit of a professional identity that includes further developing their praxis-orientation. This praxis-orientation forms part of students’ personal epistemologies and thereby motivates their engagement in practices for learning and contributes to how such practices manifest in the placement sites. Feeling valued and as if contributing to the good of the organisation and community is aligned with these students’ motivations to make a positive impact as professionals and supports the transformative potential of the placement. The TRPM placements also positively reinforced how learned practices in similar sites or with similar clients can enable students to achieve this goal and thereby contribute to better health outcomes, particularly for rural people. This finding demonstrates what Hopwood describes as the most valuable capacity of the TPA; “when we use it to discern—even if only in glimpses and suggestions—praxis, critique and envisioning of futures” reflective of “political and ethical commitments” of the agentic learner and others coming to practice [4] (p. 82).

Specifically, this research identified socio-political arrangements that supported the unique forms of relating by students to their supervisors, other professions, the consumers, and the community which concomitantly fostered students’ further development of professional practice as praxis. The TRPM required students to be highly autonomous. The autonomy given to students was supported by different power dynamics than students were accustomed to on traditional placements. Placements have been identified as environments where there is significant power held by the placement supervisor [37,38]. The TPRM created an environment where power was distributed to the student and the community by allowing these stakeholders to determine the project and activities undertaken during the placement. Other research has demonstrated that the way to facilitate the development of student agency is to position them as co-producers with staff to develop and evaluate learning [39]. This was reflected in the TRPM through workplace arrangements and associated practices that enabled students to feel as insiders and as part of the team, as well as giving students in the service-learning placements co-ownership over their project. A review undertaken by Perry et al. [40] demonstrated that valuing and respecting student nurses promotes belongingness and empowers students to engage with learning. The shared socio-political arrangement of placements supported students to gain similar learnings, including of rurally based practice potentials, across different practice sites.

The spread of student supervision across different professions also influenced the students’ agency, perceptions of value and capacity to access different opportunities for learning. Although the students were ultimately assessed by a qualified member of their profession, their day-to-day activities were undertaken as part of a larger, interprofessional team. This created a situation where students viewed themselves as being part of an organisation. Olupeliyawa et al. [41] have previously commented on the ability of interdisciplinary teaching to act as a social-political enabler for the growth of medical careers by fostering collaboration and changing the power dynamics present in teaching. Although they were examining medical professionals in their study [41], this does point to the potential importance of using interdisciplinary learning as a means of empowering the learner. This is worthy of exploration in future research.

While the uniqueness of the TRPM placement design created arrangements that some students immediately recognised as affordances for their learning, for others the placement design presented a significant challenge. This was widely attributed to the placement design’s unfamiliarity, which, for some students, initially operated as a barrier to their engagement in the placement. The assistance of the placement supervisor, peers, facilitators, and the students’ eagerness to participate in the process helped the students overcome this difficulty. Although initially a constraint, this challenge fostered student learning through problem-solving. Hopwood et al. have described this process as “problem-solving in actu” [42] (p. 16); where, in their study, health professionals learnt through doing; producing ‘brilliant practices’ in an interdisciplinary environment by being challenged and collectively problem-solving. This was enabled through distinct physical and relational arrangements, also made possible by a shared moral drive for the “good” of the individual client and their family [42] (p. 18). Salam et al. [43] have also described how challenging students through non-traditional placement allows students to develop new skills and realise other benefits such as development of independence, critical thinking, and a sense of giving back to the community. The findings of the TRPM research similarly indicate that important mediators of learning from challenges are social-political arrangements such as accessible support (peers, supervisors, other professionals), underpinned by a praxis-orientation.

Student placement preparation is also key. Other researchers have described pre-placement preparation as a fundamental component of a positive WIL experience [44,45,46]. Pre-placement preparation primes students for the psychological and emotional demands of placement [47]. It may also place students in a mindset that allows them to enter the workplace ready to engage with learning opportunities [48]. Reflecting this preparedness for learning, Campbell et al. [49] have drawn parallels between pre-placement preparation and high-quality student experiences. The findings from this research are therefore coherent with the literature and point to the importance of student preparation in all instances of WIL.

The relationships between people and professions that were promoted by the TRPM design placed the students in a position where they had access to learning from several sources. This emphasises the importance of arrangements such as placing students in pairs, optimising their access to clients while prioritising the client perspective, and scaffolding their supervision. Peer-learning during clinical placements has previously been identified to have benefits such as improved student confidence, teamwork skills, provision of support, development of peer feedback skills, and to generally promote the learning process [44,50,51,52]. Placing students in pairs may also have additional benefits in the context of rural placements, such as the prevention of loneliness and isolation [53]. The potential benefits of placing students in pairs are therefore multifactorial and worthy of consideration for those designing WIL opportunities. Similarly, providing access to learning from interprofessional groups has been extensively supported by research [54,55,56], with the findings from the present study indicating ‘interprofessional’ should be considered broadly.

While the TRPM placement settings were varied, a common experience for students was feeling embedded in the community. This was identified by the students as an important arrangement for their learning as it exposed them to the social, political, and discursive practices of their host site, enabled them to make meaningful contributions, and gain an authentic understanding of the transformative potential of their current and future practices in such sites. As one of the aims of the TRPM is to grow the rural health workforce, this is an important transformational affordance of the experience. Forming a sense of belonging and place attachment has been recognised as a way to support successful adjustment to a new environment [57]. The TRPM design reflects this and emphasises the importance of place and building social and community connections by supporting students to immerse themselves in the community. This placement experience equipped students with an increased awareness of how they could have a positive impact in a rural community. This also demonstrates how, with the right conditions of possibility, including a praxis-orientation, being embedded in a rural community for a relatively short period of time can still allow students to realise transformative potentials of rural placements.

## 5. Limitations

A key limitation of this study is that the students motivated to participate in this research project may have been more likely to view their experience positively, and relatedly, be autonomously motivated learners. As such, the experiences of students who may not have found the placement similarly valuable are not directly represented. By critically examining the experience of participating students, however, this research has identified and accounted for the importance of learner agency and a praxis-orientation aligned with the transformative goals of the placement as a prefiguring, but not determining, factor. A thorough description of the placement design has been referenced to allow readers to determine the applicability of the results to their setting. The researchers attempted to mitigate bias through interviews being conducted by members of the research team not involved in the establishment or coordination of the placements. It is, however, acknowledged that the researchers’ positions have potential to introduce social desirability bias into students’ accounts. This research only explored the student learning experience and therefore cannot provide evidence regarding the experience of the supervisors and the organisations and communities that hosted these placements. These stakeholder experiences are important considerations worthy of further research to demonstrate the value of the TRPM.

## 6. Conclusions

This research used the TPA as a theoretical framework to examine the impacts of rural WIL structured under the TRPM on the student learning experience. The findings demonstrate three themes: learning affordances related to placement design, learning through relationships between people and professions, and learning through rural embeddedness. These themes exemplified the importance of placement design for student learning, particularly in relation to providing placement preparation and access to multiple sources of learning such as supervisors, peers, community members. Being embedded in rural communities gave the students access to several arrangements that fostered learning, particularly through the sayings, relatings and doings that the students engaged with. This research demonstrates the transformative potential of rural WIL opportunities for learning and future rural practice.

## Figures and Tables

**Table 1:** Placement sites, type of placement, and participant characteristics of those involved in this research.

Site	Placement Type and Supervision Model	Student Details	Key Activities of Students	Data Collected
Early childhood centre	Service learning Supervised by a discipline supervisor remotely	Two 4th year physiotherapy students (5-week placement)	Shadowing early childhood educators, learning about paediatric physiotherapy practice, developing a resource to enhance educators’ ability to implement programs which improve children’s core body strength.	Interviewed in pairs
Disability support service	Service learning Supervised discipline supervisor in person	One 3rd year speech pathology student (3-week placement)	Shadowing team members, learning about speech pathology in the context of a disability service, developing a communication project resource.	Individual interview
Drug and alcohol addiction and rehabilitation service	Mental health and disability	Two 3rd year paramedicine students (3-week placement)	Shadowing different team members from a range of disciplines, communicating with patients, learning about dependence, withdrawal and detox services and the impact of drug and alcohol addiction on individuals, families, and communities.	Interviewed in pairs
Aged Care and Disability service	Mental health and disability	One 3rd year paramedicine student (3-week placement)	Shadowing different team members from a range of disciplines including diversional therapy and nursing, communicating with older patients, and learning about the needs and support available for older persons and people with mental illness. Participating in care activities and delivering diversional therapy to residents.	Individual interview
Disability Service	Mental health and disability	One 4th year physiotherapy student (5-week placement)	Shadowing different team members from a range of disciplines including Physiotherapists, Speech Pathologists and Occupational Therapists, communicating with patients, learning about paediatric physiotherapy practice in a disability service.	Individual interview

## Appendix A. Sample Interview Questions

- Overall, tell me about how the placement went?
- What element of this placement did you find most enjoyable? Can you tell me about some challenging aspects? Why do you think this was?
- How prepared did you feel for the placement prior to commencing? What could be done to change/alter this?
- Tell me about the site you went to—what is the main purpose of the service? How does the service operate/work?
- Tell me a bit about the town—what did you think? What did you like or dislike?
- If you had to prepare a peer to do this placement, what would you do or say or suggest they do to best prepare?
- What knowledge and skills do you think you brought to the service? What knowledge and skills are you taking away as a result of this placement?
- What sorts of interactions did you have with others in the community? Any interesting stories you’d like to relate?
- Can you tell me about a time on placement when you collaborated with someone outside of your discipline group to achieve a patient goal/outcome? How did that come about? What was that experience like?
- Tell me about your experience with the supervisor. What aspects of this relationship worked or need refining?
- What other suggestions do you have for this model of placement and potential future student learning opportunities?
- How could the placement and model be improved for future offerings?
